# High resolution voltammetric and field-effect transistor readout of carbon fiber microelectrode biosensors†

**DOI:** 10.1039/d2sd00023g

**Published:** 2022-04-05

**Authors:** Whirang Cho, Harmain Rafi, Seulki Cho, Arvind Balijepalli, Alexander G. Zestos

**Affiliations:** Department of Chemistry, American University Washington, D.C. 20016 USA zestos@american.edu; Biophysical and Biomedical Measurement Group, Microsystems and Nanotechnology Division, National Institute of Standards and Technology Gaithersburg 20899 USA arvind.balijepalli@nist.gov

## Abstract

Rapid and sensitive pH measurements with increased spatiotemporal resolution are imperative to probe neurochemical signals and illuminate brain function. We interfaced carbon fiber microelectrode (CFME) sensors with both fast scan cyclic voltammetry (FSCV) and field-effect transistor (FET) transducers for dynamic pH measurements. The electrochemical oxidation and reduction of functional groups on the surface of CFMEs affect their response over a physiologically relevant pH range. When measured with FET transducers, the sensitivity of the measurements over the measured pH range was found to be (101 ± 18) mV, which exceeded the Nernstian value of 59 mV by approximately 70%. Finally, we validated the functionality of CFMEs as pH sensors with FSCV *ex vivo* in rat brain coronal slices with exogenously applied solutions of varying pH values indicating that potential *in vivo* study is feasible.

## Abstract 2

Highly sensitive CFMEs as a pH sensor in tandem with both FET and FSCV methods having *ex vivo* sensing capability is demonstrated.

## Graphical abstract

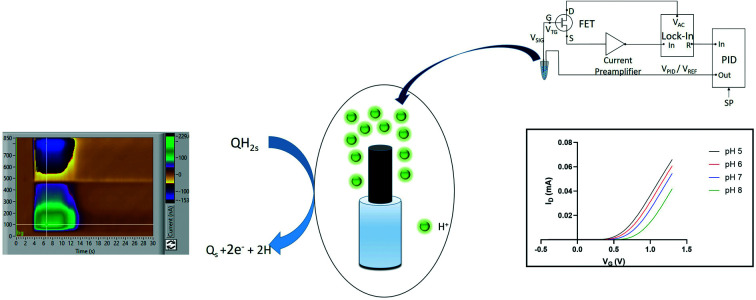

Monitoring the local, transient pH changes in the brain is gaining more attention due to its importance in understanding the functioning of brain tissue under both physiological and pathological conditions.^1–4^ For example, oxygen and pH are coupled through blood flow and metabolism because of transient neural activity.^5^ Significant pH changes have also been observed in extracellular tumor microenvironments.^6,7^ The reduced footprint of electrochemical microsensors make them well suited for *in vivo* measurements, enabling diagnostic applications in cancer studies.^5,8–12^ Fast scan cyclic voltammetry (FSCV) with carbon fiber microelectrodes (CFMEs) offers a unique capability to detect target neurotransmitters by rapidly oxidizing and reducing electroactive species at the electrode surface.^13–17^ The small size and biocompatibility of CFMEs in conjunction with excellent spatiotemporal resolution impart minimal tissue damage, thus enabling the measurement of pH in the brain *in vivo* over a relatively long time period.^18–22^ While FSCV offers chemical selectivity and the capability to distinguish co-released electroactive molecules, the challenge remains in integrating other customized transduction elements to improve the resolution, sensitivity, selectivity and other performance parameters needed for various applications.^4,12,23–25^

Biosensors based on field-effect transistors (FETs) that operate in a remote configuration allow for diverse pH sensitive films to be tested.^25–28^ FET sensors have been used as effective biosensors for several biomolecules^29^ including dopamine,^30^ phenylalanine,^7^ DNA/RNA,^31^ cortisol,^32^ serotonin, sphingosine-1-phosphate (S1P), and glucose. Glass pH probes are commonly used for pH measurements,^33,34^ however they suffer from drift, limited storage in a wet environment, frequent recalibration, and interference from alkali metals. Furthermore, amperometric measurements cannot easily distinguish electroactive molecules that are co-released due to little chemical selectivity,^35^ therefore, additional voltammetric studies coupled with FSCV represent a promising approach for combining best aspects of these techniques. Here, we integrate CFMEs with FETs to take advantage of their scalability and capacity for high resolution measurements.

For *ex vivo* biomolecule measurements, most solutions are buffered, and their pH is adjusted to the physiological value of 7.4. Any changes to the pH of the solution from this baseline physiological value will result in signal shifts. The potentials of an electrochemical reaction show the propensity of an electroactive species to accept and donate electrons through oxidation/reduction reactions in addition to electron-transfer kinetics and analyte mass-transport. Therefore, peak oxidative currents in cyclic voltammograms (CV) are related to specific faradaic redox processes resulting in a chemical-specific “fingerprint”.^36^ CV features for pH changes originate from redox reactions of electrochemically active surface groups, such as phenols, *ortho*- and *para*-quinones, carbonyls, lactones, and carboxylic acids on carbon electrode surfaces.^37^ CFMEs have the efficacy of the fast, biocompatible, spatially resolved sensitive, and selective pH sensors both *in vitro* and *in vivo*.

In this study, we have developed the use of CFMEs as pH sensors using both FET and FSCV transduction. CFMEs were sensitive to changes within the physiological range of pH 5–8. When measured with standard pH buffers, the measurement of sensitivity of CFMEs was found to be consistent with the Nernst value of ≈59 mV for the FET setup, while they were found to have a current sensitivity of (173.0 ± 8.2 nA) with the FSCV-based measurements, where the error bar represents standard error in the current. Proof of principle work was performed with mouse coronal brain slices where several pH solutions were exogenously applied and measured with FSCV on CFMEs. It shows that CFMEs are also sensitive to pH changes in biological tissue such as brain slices. This new application will potentially create novel pH sensors using FSCV and FET methods for *ex vivo* and *in vivo* measurements.

The transfer properties of the FET were measured by recording drain current (*I*_D_) as a function of gate potential (*V*_G_) while keeping drain voltage (*V*_D_) constant. In this measurement, I_D_ measured as a function of *V*_G_ with CFMEs compared to a glass pH probe measuring standard buffer solutions with pH 2, 4, 7, and 10 were sequentially connected to the FET as shown in Fig. S1.† The measurement sensitivity of CFME (≈58 mV) was slightly higher than that of glass pH probe (≈50 mV) and consistent with the theoretical Nernst value of ≈59 mV at room temperature.^38^ The sensing of pH is based on the protonation and deprotonation of hydroxyl groups on the sensor surface and its subsequent transduction by the FET gate. Under acidic conditions, surface OH group tends to protonate as OH_2_^+^, which leads to an increase in the effective surface potential, resulting in larger *I*_D_. On the other hand, under basic conditions, the deprotonation of OH group produces O^−^ surface charge that reduces the surface potential and leads to a decrease of *I*_D_. Therefore, pH signals can be converted into electrical signals through the FET transducer.^39,40^

CFMEs can also be effectively used for measuring the pH of aCSF (artificial cerebrospinal fluid) buffer solutions with high sensitivity. Fig. 1(b) shows the change in the gate voltage (*V*_G_) for a representative sample measured within the range of pH 5 to pH 8. The normalized average *V*_t,G_ exhibited good linearity (*R*^2^ = 0.884) as shown in Fig. 1(c) resulting in a sensitivity of (101 ± 18 mV), ≈70% higher than the Nernst value of 59 mV (*n* = 3). The reported uncertainty corresponds to the standard error of the slope of the fit in Fig. 1c. Measurements with three independent electrodes are shown in Fig. S2.†

**Fig. 1:**
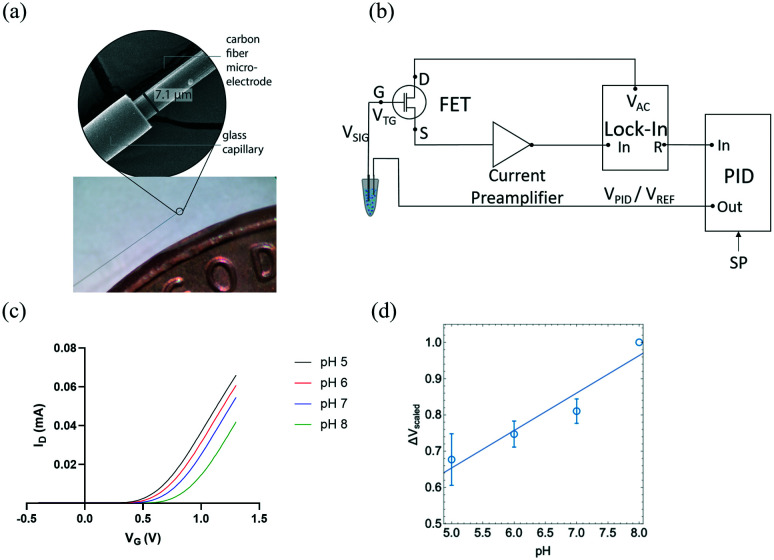
(a) SEM and optical microscope images of carbon fiber microelectrodes (CFMEs) (b) field effect transistors (FET) measurement schematic of closed-look pH measurements using proportional-integral-derivative (PID) coupled with narrowband detection using a lock-in amplifier (adapted with permission from [*Analyst* 2020, 145, 2925–2936]. Copyright [2020] [Royal Society of Chemistry]). (c) Representative change in the gate voltage (*V*_G_) as a function of aCSF buffer solution pH, which shows (d) a linear relationship between pH and *V*_G_ (*n* = 3). The pH sensitivity, determined from the slope of the curve was (101 ± 18 mV), where the error bar represents the standard deviation of each measured data point.

Here for the first time, we use carbon fiber microelectrodes (CFMEs) paired with FET transducers for the measurement of pH. The small sizes of CFMEs (≈7 μm in diameter) ensures specific targeting of specific *in vivo* sub-regions when implanted. High spatiotemporal resolution (<10 ms) of CFMEs allows for the fast measurements of transient changes in pH. Moreover, CFMEs are carbon-based and, therefore, less prone to the adsorption of oxidation by-products and fouling, enabling their use within biological tissue.^24,35,36^

We demonstrate the use of CFMEs as working electrodes to measure transient pH changes in the flow cell *in vitro* to allow direct comparisons with the FSCV measurements. To measure redox behaviour of CFMEs as a function of varying pH, a bi-directional triangle waveform was applied to the electrode over a potential range of −0.4 V to 1.3 V at a scan rate of 400 V s^−1^ and a CV sampling rate of 10 scans per second. The oxidation of a hydroquinone-like moiety on the surface of bare CFMEs^20^ occurs at 0.6 V during the forward scan (Fig. 2a; A) followed by reduction at −0.18 V on the backward scan (Fig. 2a; B). The false color plot shows distinction between positive oxidative current and negative reduction current. In addition, as shown in Fig. S3,† the square shaped peak oxidative current at 0.6 V *vs.* time (*I vs. T*) traces show the high temporal resolution of the redox reaction with the hydroquinone-like moiety. The current around −0.1 V can be attributed to double layer charging, which originates from non-faradaic processes.^20^

**Fig. 2:**
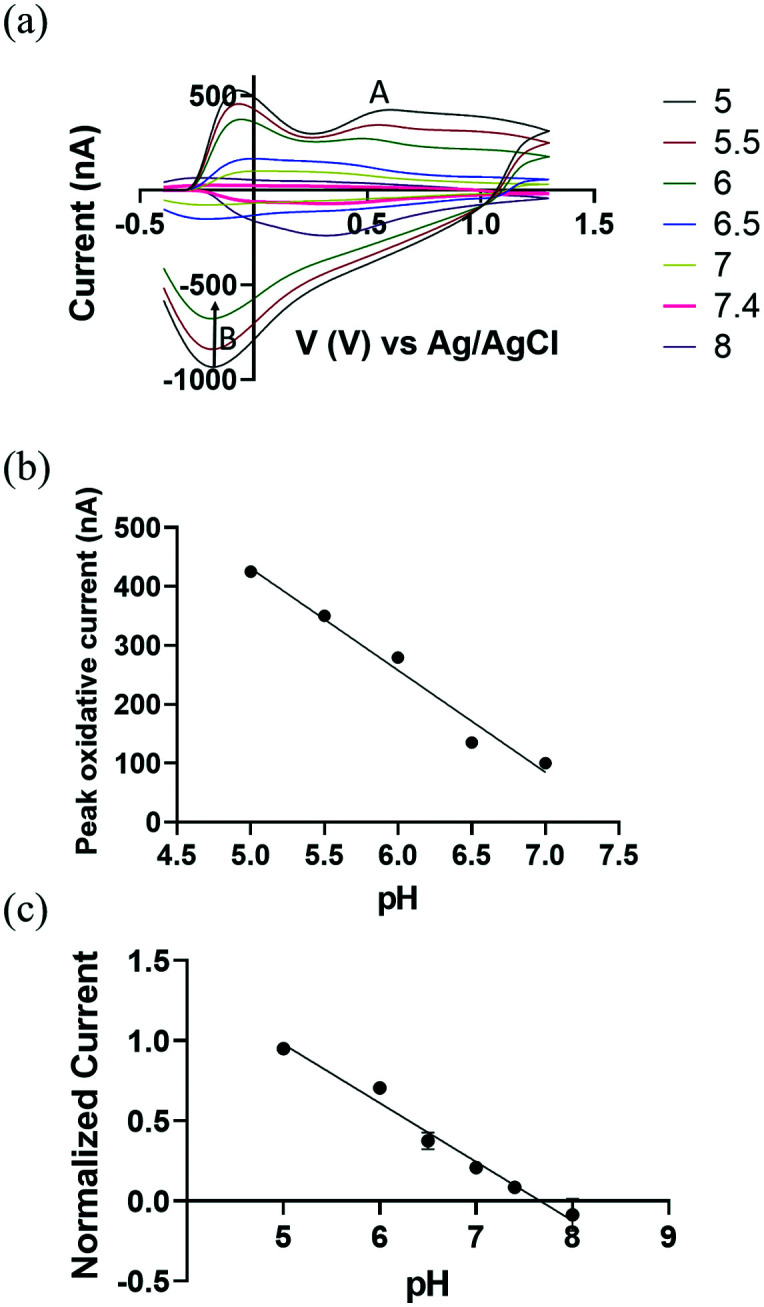
(a) Background subtracted cyclic voltammograms (CV) as a function of pH changes ranged from 5 to 7 (a triangle waveform was applied from −0.4 V to 1.3 V and back at a scan rate of 400 V s^−1^ and a frequency of 10 Hz). (b) A linear relationship between pH and peak oxidative current (A peak, nA). *R*^2^ = 0.953 (c) the normalized average peak oxidative current as a function of pH (*n* = 5), *R*^2^ = 0.851. The error bars in (b) and (c) represent the standard deviation for each value of pH. The error bars are smaller than the symbols in the curves.

We hypothesize that the presence of surface oxide groups, including quinones on the surface of the CFMEs is primarily responsible for the sensitivity to changes in pH (ΔpH). Carbon fibers were formed from graphitic carbon with a surface rich with negatively charged oxide groups.^41^ Applying a voltage waveform to the CFMEs breaks carbon–carbon bonds within the fibers, increases surface roughness, and further functionalizes CFMEs with oxide containing groups, such as protonated quinones, carbonyl, hydroxyl, and carboxyl groups.^42,43^ Previous studies^44^ have shown that a polished electrode reduced the sensitivity of CFMEs to pH through the elimination of surface oxide groups. They showed that coating the electrode with an anionic cation-exchange polymer restored the responsiveness and sensitivity to pH and catecholamines while maintaining sensitivity.^44^

In Fig. 2b, we observe a linear response between pH and the peak oxidative current at around 0.6 V (Fig. 2a; A), where the slope of linear plot (sensitivity) was −(173.0 ± 8.2 nA), ≈110% higher than previously reported values, where the error bar represents the standard error of the measurement.^20^ This improved performance can be attributed to the increased length of our carbon fiber microelectrode and more abundant protonated surface bound quinones groups on the CFMEs by lowering pH down to 5. To eliminate the probable effect from miniscule difference in carbon fibers length ≈100 μm, peak oxidative current was normalized as a function of electrode length per pH (see Fig. 2c) (*n* = 5).

We then explore the practicality of CFMEs pH sensor tandem with FSCV in measuring transient changes of pH by exogenously applying different pH solutions in rat brain slices. After extracting a rat brain, it was excised to bilaterally target the caudate putamen (CPu),^46,47^ marked by the black circle in Fig. 3(a). The brain slice was placed into a 24-well plate and saturated with aCSF buffer, which was oxygenated by bubbling carbogen gas (95% O_2_, 5% CO_2_). CFMEs were then lowered until they were immersed into brain tissue and were allowed to equilibrate at least for 15 min. aCSF buffer was applied with different pH values ranging from 3 to 6 by subsequently injecting 250 μL of each pH solution into the brain slice and adjacent to the CFMEs. Injections were repeated three times at each pH with 10 min intervals between them. As shown in Fig. 3(b), the peak occurring at −0.28 V originates from the redox reaction at the surface-bound hydroquinone-like moiety. As with the *in vitro* data, basic pH values (up to 6) decreased the overall peak oxidative current at ≈−0.28 V and slightly changed the shape of the CV curves. O_2_ can also play a role in changing pH, however O_2_ reduction occurs near −1.3 V. Therefore, the pH contribution from O_2_ does not affect the measurement.^8,18,48^ The peak oxidative current exhibited a linear response between pH ranging from 3 to 6, where the slope (sensitivity) was ≈−7 nA (Fig. 3c).

**Fig. 3:**
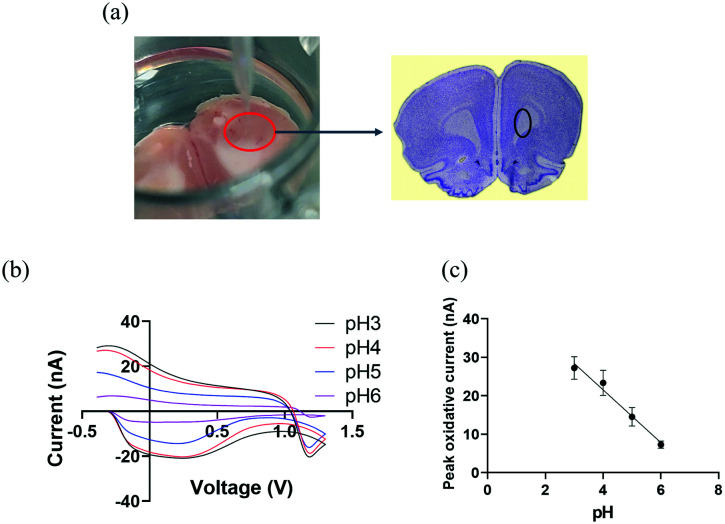
(a) *Ex vivo* experimental set-up: working electrode (CFMEs) and rat brain slice were place in a 24-well plate and the reference electrode (Ag/AgCl) was placed adjacently to the CFMEs into the brain slice. The rat brain atlas was adapted from Allen Mouse Brain Atlas (2004) and the Bregma 3.24 mm, Paxinos and Watson Atlas^45^ (b) background subtracted cyclic voltammograms (CV) as a function of pH changes ranged from 3 to 6 recorded in a rat brain slice (a triangle waveform was applied from −0.4 V to 1.3 V and back at a scan rate of 400 V s^−1^ and a frequency of 10 Hz). (c) A linear relationship was observed between pH and peak oxidative current (*R*^2^ = 0.913). The error bars represent the standard deviation for each pH.

The difference in CV shape and sensitivity between the *in vitro* and the *ex vivo* measurements can be attributed to presence of blood, proteins, and other molecules in the coronal brain slice tissue, which can result in non-specific interactions with the CFMEs. These interactions could alter the CFMEs response relative to bare, unmodified microelectrodes. However, despite the change in CV shape, not only are they reproducible from sample to sample but CFMEs are also still highly sensitive to pH changes when immersed in biological tissue such as brain slices. We have shown that these measurements can be made *ex vivo* in brain slice biological tissue, which shows that real samples do not interfere or prevent the measurement of pH. These measurements illustrate proof of principle studies that CFMEs can indeed measure pH changes with FSCV when immersed into biological tissue such as coronal brain slices.

## Conclusions

In summary, our study opens a new opportunity for CFMEs pH sensor with high sensitivity integrated with both FSCV and FET. We have measured exogenously applied pH changes *ex vivo* in mouse brain tissue, which illustrates that potential *in vivo* studies are indeed feasible. The sensitive, fast, biocompatible, and selective detection of fluctuations of pH provides for a multitude of potential future applications such as the optimization of biomolecule measurement and measurement of pH in extracellular tumor microenvironments for cancer studies in addition to many others, which makes this a significant study.

We would like to acknowledge partial funding from the following sources: American University Faculty Research Support grant (A. G. Z.), the Faculty Mellon Grant, the NASA DC Space grant, NIH 1R41NS113702-01 (A. G. Z.), the SACP Pittcon Starter grant, and the NSF I-Corps #1936173 (A. G. Z.).

All animal procedures were performed in accordance with the Guidelines for Care and Use of Laboratory Animals of American University and approved by the Animal Ethics Committee of the Institutional Animal Care and Use Committee (IACUC) of American University and Protocol #20-09.

## Author contributions

Whirang Cho: conceptualization, investigation, data curation, visualization, writing – original draft, review & editing. Harmain Rafi: investigation. Seulki Cho: investigation, software. Arvind Balijepalli: conceptualization, investigation, data curation, software, visualization, writing – review & editing, supervision. Alexander G. Zestos: conceptualization, visualization, investigation, writing – review & editing, funding acquisition, supervision.

## Conflicts of interest

There are no conflicts to declare.

## Supplementary Material

SD-001-D2SD00023G-s001
