# Correction: High resolution voltammetric and field-effect transistor readout of carbon fiber microelectrode biosensors

**DOI:** 10.1039/d5sd90047f

**Published:** 2025-12-09

**Authors:** Whirang Cho, Harmain Rafi, Seulki Cho, Arvind Balijepalli, Alexander G. Zestos

**Affiliations:** a Department of Chemistry, American University Washington, D.C. 20016 USA zestos@american.edu; b Biophysical and Biomedical Measurement Group, Microsystems and Nanotechnology Division, National Institute of Standards and Technology Gaithersburg 20899 USA arvind.balijepalli@nist.gov

## Abstract

Correction for ‘High resolution voltammetric and field-effect transistor readout of carbon fiber microelectrode biosensors’ by Whirang Cho *et al.*, *Sens. Diagn.*, 2022, **1**, 460–464, https://doi.org/10.1039/D2SD00023G.

The authors regret that a statement confirming the use of live subjects in the study was incomplete in the published article and supplementary information. This complete statement is detailed below.

All experiments were conducted in accordance with the relevant laws and institutional guidelines applicable to this work. Tissue samples were collected from female Sprague Dawley rats in accordance with the Institutional Animal Care and Use Committee (IACUC) and animal facility protocols at American University and Protocol #20–09.

The Royal Society of Chemistry apologises for these errors and any consequent inconvenience to authors and readers.

