# Supplementary material for: High resolution voltammetric and field-effect transistor readout of carbon fiber microelectrode biosensors
Source: Sens Diagn. Author manuscript; Available in PMC 2022 May 26. (PMC9119317; doi:10.1039/d2sd00023g)
Supplement: Supplementary Information [file NIHMS1797942-supplement-Supplementary_Information.pdf]

Electronic Supplementary Material (ESI) for Sensors & Diagnostics.
This journal is © The Royal Society of Chemistry 2022

Supplementary Information

High Resolution Voltammetric and Field-Effect Transistor Readout of Carbon Fiber Microelectrode Biosensors

Whirang Cho[1,2], Harmain Rafi[1], Seulki Cho[2], Arvind Balijepalli[2,*], Alexander Zestos[1,*]

[1]Department of Chemistry, American University, Washington, D.C. 20016, United States

[2]Biophysical and Biomedical Measurement Group, Microsystems and Nanotechnology Division, National Institute of Standards and Technology, Gaithersburg 20899, United States

Experimental

Chemicals and Reagents: Artificial cerebrospinal fluid (aCSF) was prepared (145 mmol/L NaCl, 2.68 mmol/L KCl, 1.4 mmol/L $CaCl_2$, 0.45 mmol/L NaH_2PO_4 1.01 mmol/L $MgSO_4$, and 1.55 mmol/L Na_2HPO_4) with the pH adjusted to 7.4. Acidic and basic solutions were adjusted to various pH values with the addition of HCl and NaOH, respectively. All aqueous solutions were made with deionized water.

CFME Preparation. The CFME preparation was based on previously reported procedures.[1,2] Briefly, a single strand carbon fiber of 7 μm in diameter was aspirated into a glass capillary with a 1.2 mm outer and 0.68 mm inner diameter using a vacuum pump. Carbon fibers were pulled to form two electrodes on a vertical pipette puller and then cut to lengths of approximately 100 μm. To stabilize the carbon fiber inside the glass capillary and prevent leakage of backfilled saturated

KCl solution, protruding CFME tips were epoxied and then rinsed in acetone to wash away any excess residual epoxy. The electrodes were cured in the oven for 4 h at 125 °C.

Scanning Electron Microscope (SEM). The thickness and morphologies of CFMEs were obtained with a JEOL JSM-IT100 (JEOL, Tokyo, Japan) at a 10 kV accelerating voltage.

Field Effect Transistor (FET). We fabricated a commercially sourced single-gate FET combined with signal processing to significantly improve the overall performance of the complete sensor system. Here, lock-in amplifier (LIA) was widely used to improve the overall signal-to-noise ratio (SNR) by allowing the recovery of weak signals at a specific reference frequency and phase. The pH resolution of FET can be further increased by integrating LIA to recover weak signals. For the measurement, CFMEs applied to the top gate modulate the current in the semiconducting channel of FET. An Ag/AgCl reference electrode was connected to the output of PID (proportional–integral–derivative) controller, and it was used to adjust the gate potential and maintain a constant channel current. PID controllers are widely used to control process variables for increased stability and accuracy by continually adjusting a process parameter in response to deviations from a predetermined set point. The PID is commonly used in controlled temperature sensors, gas detectors, photosensors, and hydrogen sensors. Moreover, it is also applied to improve the reproducibility and accuracy of atomic force microscopy (AFM) by controlling gain parameters.

Fast-Scan Cyclic Voltammetry. The cyclic voltammograms were obtained using WaveNeuro FSCV System with 5 MΩ headstage (Pine Instruments)[1]. For data collection and analysis, the software high-definition cyclic voltammetry with PC1e-6363 multifunction I/O device (National Instruments, Austin, TX) was used. Upon testing an electrode, a triangle waveform was applied

[1] Certain equipment and materials are identified in this paper in order to specify the experimental procedure adequately. Such identification is not intended to imply endorsement by the National Institute of Standards and Technology, nor is it intended to imply that the materials or equipment identified are necessarily the best available.

with a holding potential of −0.4 V scanning up to 1.3 V against the silver−silver chloride reference electrode (Ag/AgCl, −0.197 V) and back at a scan rate of 400 V/s with a frequency of 10 Hz. Each electrode was back-filled with 0.1 mol/L KCl solution to create an electrical connection and was allowed to equilibrate with the applied waveform at least for 10 min. Samples were tested in a flow injection analysis system (In Vitro/FSCV Microelectrode Flow Cell with xyz micromanipulator Translational Stage, Pine Instruments, Durham, NC). The buffer and samples were pumped through the flow cell at 1 mL/min using a NE-300 Just Infusion Syringe Pump (New Era Pump Systems, Farmingdale, NY). For the measurement, the electrode is placed into a custom-made flow cell and buffer is flowed past the electrode surface for approximately 5 seconds while the waveform is applied. At that time, a solution of aCSF at a specific pH is injected with a syringe and flowed past the electrode surface with the data being collected for an additional 10 seconds. Cyclic voltammograms (CVs) are background subtracted to separate the background charging (non-faradaic) current from the faradaic current. Data are shown in the form of CVs, current vs. time traces (I vs. t) and false three-dimensional color plots.

Redox equation of the hydroquinone-like molecule.

$$QH_{2s} \rightleftarrows Qs + 2e^{-} + 2H \quad (1)$$

where QH_{2s} and Q_s indicate the reduced and oxidized form of a surface hydroquinone-like moiety. QH-peak shows the transition for the hydroquinone to quinone on the forward scans while Q-peak indicates the transition for the quinone to hydroquinone on the backward scans.[3]

Tissue Extraction. Tissue samples were collected from female Sprague Dawley rats in accordance with IACUC and animal facility protocols at American University (Protocol #20-09). Rats were housed in 12 hour light and dark cycles and provided food and water *ad libidum*. Firstly, a rat was removed from the cage and placed in a CO_2 euthanasia chamber. Reflexes of a rat were then assessed via toe and tail pinch, euthanasia was subsequently confirmed by cervical dislocation. The head was decapitated using surgical scissors and the skull was exposed by removing surrounding tissue. Large, surgical rongeurs were used to quickly peel away the skull bones to expose and remove the brain. The excised brain was placed into a vial filled with artificial cerebrospinal fluid (aCSF) and stored on ice until use. For slice preparation, multiple coronal cuts were made to target either the caudate putamen or the hippocampus. Coordinates and anatomical landmarks were located according to the Paxinos Rat Brain Atlas.[4]

Brain tissue preparation procedures were adapted from Papouin and Haydon.[5] The brain slice was placed into the well of a 24-well plate (Castar, Corning, New York). The slice was then saturated with a cold aCSF buffer, which had been oxygenated by bubbling carbogen gas (95 % O_2, 5 % CO_2) using an air stone and airline tubing. Subsequently, CFMEs was lowered until it made a contact with the brain tissue and was allowed to equilibrate at least for 15 min with a triangle waveform. aCSF buffer solutions having different pH ranged from 2 to 8 were then exogenously applied by injecting 250 µL of each solution into the slice and adjacent to the CFMEs. After each injection, the residual aCSF solution in the 24-well plate was discarded and a fresh, oxygenated aCSF was added. Injections were repeated 3 times at each pH with 10 min intervals between them.

References

[1] R. Dilpreet, A. Mendoza, P. Wonnenberg, S. Mohanaraj, M. Sarbanes, C. Truong, and A. G. Zestos, *Analytical Methods*, 2019, 11, 1620-1630.

[2] T. Asrat, W. Cho, F. A. Liu, S. M. Shapiro, J. R. Bracht, and A. G. Zestos, *ACS omega*, 2021, 6, 6571-6581.

[3] P. Takmakov, M. K. Zachek, R. B. Keithley, E. S. Bucher, G. S. McCarty and R. M. Wightman, Analytical chemistry, 2010, 82, 9892-9900.

[4] G. Paxinos and C. Watson, The Rat Brain in Stereotaxic Coordinates: Hard Cover Edition, p. 451, Elsevier, (2006).

[5] T. Papouin, P. G. Haydon, Obtaining Acute Brain Slices. Bio-Protocol, (2018).

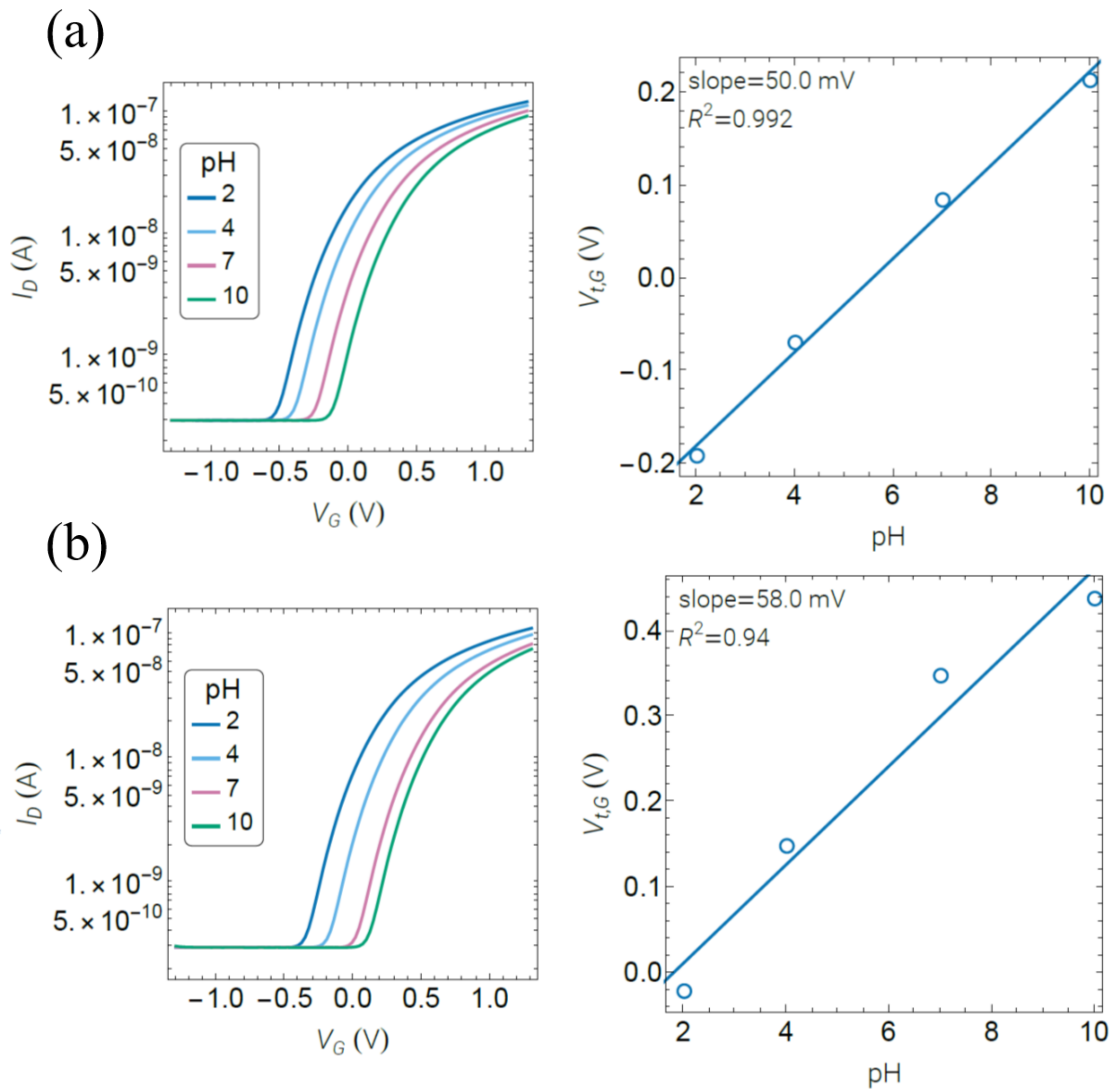

Figure S1. Change in the back-gate threshold voltage (V_{BG}) as a function of standard pH buffer solution, which shows a linear relationship between pH and V_{BG}. (a) Glass electrode probe showing pH sensitivity of 50.0 mV/pH (b) CFMEs showing pH sensitivity of 58.0 mV/pH. (uncertainties are smaller than symbol size)

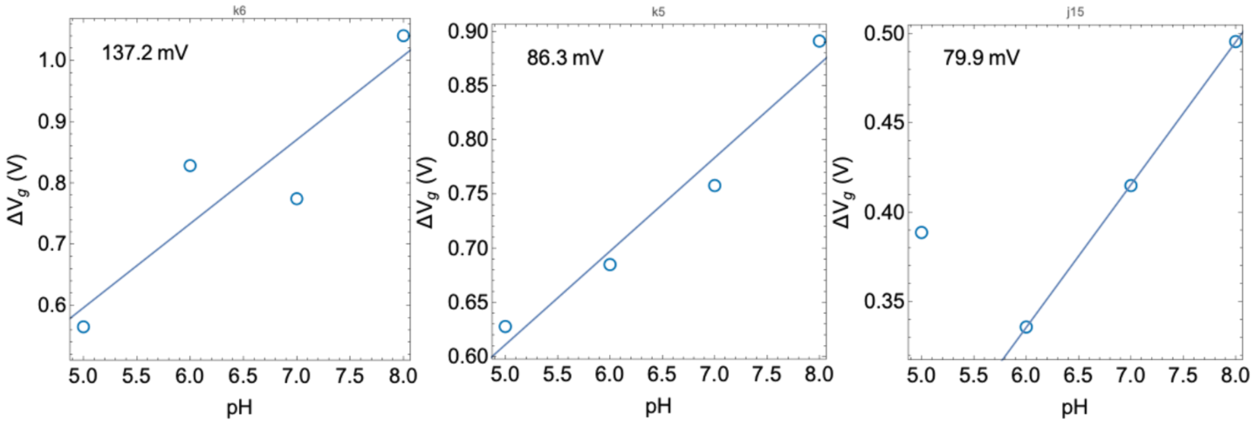

Figure S2. The changes in the gate threshold voltage ($\Delta V_{t,G}$) as a function of pH showed a linear response over the measured pH ranging from 5 to 8. (uncertainties are smaller than symbol size)

(a) (b)

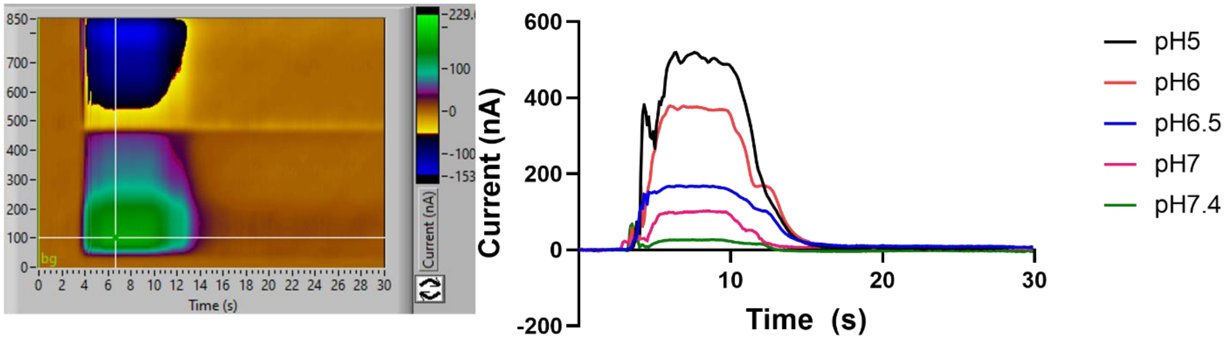

Figure S3. (a) Representative false color plot for aCSF buffer solutions of exogenously applied pH 6.5 (b) I (current) vs T (time) trace as a function of pH changes.
